# Changes in the acceptance of artificial intelligence in medicine among physicians and the general public in Japan: A repeated cross-sectional comparison of 2018 and 2025

**DOI:** 10.1371/journal.pdig.0001769

**Published:** 2026-10-23

**Authors:** Teppei Suzuki, Honoka Tamori, Kazuki Ohashi, Yasuhiro Morii, Kenji Hirata, Kohsuke Kudo, Katsuhiko Ogasawara

**Affiliations:** 1 Iwamizawa Campus, Hokkaido University of Education, Iwamizawa City, Hokkaido, Japan; 2 Faculty of Health Sciences, Hokkaido University, Sapporo, Japan; 3 Center for Outcomes Research and Economic Evaluation for Health, National Institute of Public Health, Wako, Saitama, Japan; 4 Department of Diagnostic Imaging, Faculty of Medicine, Hokkaido University, Sapporo, Hokkaido, Japan; 5 Muroran Institute of Technology, Graduate School of Engineering, Muroran, Japan; University of Bremen Faculty 11 Human and Health Sciences: Universitat Bremen Fachbereich 11 Human- und Gesundheitswissenschaften, GERMANY

## Abstract

Medical artificial intelligence (AI) has advanced rapidly, yet whether growing societal familiarity with AI is accompanied by greater willingness to receive relatively autonomous AI-led care remains unclear. We conducted repeated cross-sectional online surveys in Japan in 2018 and 2025 using the same questionnaire and compared self-identified physicians and members of the general public (2018: 399 physicians and 600 members of the public; 2025: 300 physicians and 300 members of the public). Mean responses to the item assessing intention to use medical AI did not differ significantly between survey years in either group, despite consistently favorable evaluations of its usefulness and future necessity. Among physicians, the unadjusted mean response regarding the potential of medical AI to improve clinical efficiency was higher in 2025, but this difference was not significant after adjustment for age and sex. Among members of the public, self-rated knowledge of medical AI was higher in 2025, while expectations that medical AI would increase healthcare costs were also greater. These findings suggest that increased self-rated knowledge may not necessarily be accompanied by greater intention to use relatively autonomous medical AI. Implementation strategies should therefore address concerns regarding responsibility, safety, data protection, and healthcare costs alongside efforts to improve public understanding.

## Author summary

Artificial intelligence (AI) is increasingly used in healthcare, where it may support diagnosis, treatment decision-making, and clinical operations. As AI technologies have advanced and become more familiar in society, attitudes toward medical AI may also have changed. However, greater familiarity with AI does not necessarily mean that physicians or members of the public are more willing to receive care led by AI. To examine these changes, we compared two online surveys conducted in Japan in 2018 and 2025 using the same questionnaire. Both physicians and members of the general public continued to recognize the usefulness and future necessity of medical AI, but their intention to use it did not significantly differ between the two survey years. Among physicians, expectations that AI could improve clinical efficiency were higher in the unadjusted comparison, but this difference was no longer significant after accounting for age and sex. Among members of the public, self-rated knowledge of medical AI was higher in 2025, while expectations that AI would increase healthcare costs were also greater. These findings suggest that greater knowledge or exposure to AI may not automatically lead to greater willingness to use relatively autonomous medical AI. Building informed trust will require improving understanding while also addressing persistent concerns surrounding the use of medical AI.

## Background

Artificial intelligence (AI) has increasingly been introduced into healthcare to support clinical diagnosis, treatment decision-making, and operational efficiency [1,2]. However, acceptance of medical AI has remained uncertain, particularly because patients and the public have expressed concerns regarding safety, accountability, and the role of AI in clinical care [3]. In Japan, government-led initiatives promoting the societal implementation of medical AI began around 2017, increasing the need to understand attitudes among both healthcare professionals and the general public. Against this background, Tamori et al. conducted a nationwide survey in 2018 and found that physicians were generally more positive about medical AI and reported greater intention to use it than members of the general public, although both groups expressed substantial concerns regarding legal responsibility and explainability [4].

Similar findings have been reported internationally. Surveys of patients and the public have shown that perceived usefulness may coexist with concerns regarding safety and accountability [5], while physicians have reported positive evaluations of the clinical usefulness of AI alongside concerns about legal responsibility and explainability [6]. Since the COVID-19 pandemic, healthcare digitalization has accelerated through the expansion of telemedicine and remote monitoring [7–11]. More recently, the emergence of large language models and generative AI has further increased public and professional exposure to AI technologies [12]. At the same time, ethical, legal, and transparency-related concerns have intensified, suggesting that acceptance depends not only on technical usefulness but also on trust and appropriate governance [13,14]. In addition, Yang et al. reported that although clinical decisions supported by generative AI were considered reasonable, physicians’ perceived competence and trust among peers could be adversely affected [15], indicating that social and psychological factors also shape acceptance.

These developments suggest that attitudes toward medical AI may have changed since 2018, particularly with respect to perceived usefulness, efficiency, reliability, legal concerns, and intention to use. However, previous studies have largely relied on cross-sectional surveys and have not examined changes using the same questionnaire across different time points. Therefore, the present study conducted a repeated cross-sectional survey in 2025 using the questionnaire originally administered by Tamori et al. in 2018. The objective was to compare item-level responses among physicians and members of the general public between 2018 and 2025 and to explore the underlying factor structure of attitudes toward relatively autonomous medical AI.

## Methods

### Study design and definition of medical AI

This study used a repeated cross-sectional design to compare responses obtained in 2018 and 2025. The 2025 survey used the same questionnaire as the 2018 “Survey on Acceptance of the Use of Artificial Intelligence (AI) in Healthcare.” In both surveys, medical AI was defined as healthcare in which AI determines diagnoses and treatment plans without requiring physician involvement. This definition was retained unchanged to ensure comparability between the two survey years.

### Questionnaire

The questionnaire consisted of two sections. The first covered respondents’ basic characteristics (e.g., sex, occupation, and age). The second measured acceptance of medical AI using 20 items developed with reference to the Unified Theory of Acceptance and Use of Technology (UTAUT) [16]. Because the UTAUT was originally designed for users of specific systems, and this study included members of the general public who were non-users, items assuming actual use experience or use frequency were excluded, and the wording was modified to assess cognitive evaluations based on hypothetical use. In addition, attitude and uneasiness were included, as previous studies have confirmed them to influence intention to use. All 20 items were rated on a five-point Likert scale (1 = strongly disagree, 5 = strongly agree). Only the item related to medical costs used a different scale (1 = decrease to 5 = increase). Some items (e.g., concerns about data leakage), were reverse-coded so that higher scores consistently indicated greater acceptance. Textbox 1 presents the full wording of the 20 questionnaire items used to assess acceptance of medical AI.

### Participants and sample selection

Participants in both surveys were recruited from a commercial opt-in online research panel maintained by Rakuten Insight, Inc., Japan. Physician status was determined based on participants’ self-reported occupation and was not independently verified. Physician participants also self-reported their medical specialty by selecting one specialty category.

The 2018 survey initially included 600 members of the general public and 400 self-identified physicians. The general public sample was divided into six age groups (15–24, 25–34, 35–44, 45–54, 55–64, and ≥65 years), with 50 men and 50 women in each group. The physician sample consisted of participants aged ≥25 years and included 350 men and 50 women. Physicians were recruited regardless of clinical experience or medical specialty. One respondent who self-identified as a physician was excluded because the reported highest educational attainment was vocational school/junior college, which was inconsistent with the educational requirements for obtaining a medical license in Japan. The final 2018 analytical sample therefore consisted of 399 physicians and 600 members of the general public.

The 2025 survey included 600 participants, comprising 300 self-identified physicians and 300 members of the general public. No formal a priori sample size calculation was conducted. The target sample size of 300 physicians and 300 members of the general public was determined based on the available research budget and the feasibility of recruitment through the commercial online panel.

### Survey procedures and ethical considerations

Before beginning the 2025 survey, participants were provided with information regarding the purpose of the study, the approximate completion time, the voluntary nature of participation, and the handling of their responses. Participants provided electronic consent before proceeding to the questionnaire. All responses were anonymous, and no personally identifiable information was collected. Participants received panel points upon completion of the survey. Information on sex, age, and highest educational attainment was collected as demographic characteristics. All analyses were conducted separately for physicians and members of the general public.

### Statistical analysis

The 2018 and 2025 samples were treated as independent samples, and responses were compared separately for physicians and members of the general public.

For each item, the mean and standard deviation were calculated and compared between 2018 and 2025. Analyses were conducted separately for physicians and the general public using independent-samples t-tests. Effect sizes were calculated using Cohen’s d and corresponding 95% confidence intervals and interpreted according to Cohen’s (1988) criteria, with values of 0.2, 0.5, and 0.8 indicating small, medium, and large effects, respectively.

Because the age distribution differed between the 2018 and 2025 physician samples, additional ordinal logistic regression analyses were conducted as sensitivity analyses. Separate models were fitted for the items assessing intention to use medical AI and the perceived potential of medical AI to improve clinical efficiency. Survey year was included as the primary explanatory variable, with age and sex included as covariates. The questionnaire responses were treated as ordinal outcomes.

Textbox 1. Question items measuring acceptance factors

**Table:** 

No.	Items	Question text
Q1	Interest in topic	Are you interested in the topic of AI in medicine?
Q2	Intention to use	Would you be willing to use AI-driven medicine?
Q3	Usefulness	Do you think that AI in medicine will be useful?
Q4	User-friendliness	Could physicians easily operate AI in medicine settings?
Q5	Necessity of legislation	Do you think the use of AI in medicine should be regulated by legislation?
Q6	Accuracy	Do you think AI in medicine will deliver accurate diagnoses?
Q7	Efficiency	If AI is used in medicine, do you think physicians could provide services more efficiently?
Q8	Relevance to life	Do you think that AI in medicine will play an important role in your life in the future?
Q9	Better medical services	Would using AI in medicine lead to better medical services?
Q10	Expectations of others	Do you think people around you are optimistic about the potential of AI in medicine?
Q11	Expectations among patients	Do you think patients are optimistic about the potential of AI in medicine?
Q12	General impression	Do you have a generally favorable impression of the use of AI in medicine?
Q13	Mastery	Could physicians quickly master the use of AI in medicine?
Q14	Necessity in medicine	Do you think that AI will be essential in medicine in the future?
Q15	Knowledge of AI in other contexts	Do you know much about the use of AI in contexts other than medicine?
Q16	Knowledge of AI in medicine	Do you know much about the use of AI in medicine?
Q17	Concern about accountability	Are you concerned about who would be accountable for any accident resulting from the use of AI in medicine?
Q18	Concern about data leakage	Are you concerned that using AI in medicine might lead to the leakage of personal data?
Q19	Brand impact	Are your views on AI in medicine shaped by the businesses (or brands of such businesses) that developed the AI for medicine?
Q20	Medical costs	How do you think AI in medicine will affect medical costs?

### Factor analysis

Exploratory factor analysis (EFA) was conducted using the 2018 dataset to identify the factor structure underlying acceptance of medical AI. To examine the validity of the identified factor structure, confirmatory factor analysis (CFA) was then performed to evaluate the model’s fit to both the 2018 and 2025 datasets. Model fit was assessed using goodness-of-fit indices and inter-factor correlations separately for each survey year. Because the questionnaire items were ordinal in nature, estimation used diagonally weighted least squares (DWLS) method. Statistical analyses were conducted using R (version 4.5.2) and JMP, and CFA was performed using lavaan package.

Comparisons between the 2018 and 2025 surveys were conducted at the level of individual questionnaire items. The study did not compare latent factor means or composite scale scores across survey years.

## Results

The 2018 survey was conducted over a three-day period from November 13–15, 2018, and included 600 members of the general public (300 men and 300 women) and 399 physicians. The 2025 survey was conducted from June 10–12, 2025, and included 300 members of the general public and 300 physicians. Participant characteristics are presented in Table 1. The distribution of self-reported medical specialties among physicians in each survey year is presented in Supplementary S2 Table.

**Table 1:** Demographic characteristics of physicians and members of the public in the 2018 and 2025 surveys.

Characteristics	2018 Physicians (n = 399)	2018 Public (n = 600)	2025 Physicians (n = 300)	2025 Public (n = 300)
Sex				
Male	349 (87.5)	300 (50.0)	250 (83.3)	150 (50.0)
Female	50 (12.5)	300 (50.0)	50 (16.7)	150 (50.0)
Age group, years				
15–24	0 (0.0)	100 (16.7)	0 (0.0)	50 (16.7)
25–34	20 (5.0)	100 (16.7)	19 (6.3)	50 (16.7)
35–44	61 (15.3)	100 (16.7)	35 (11.7)	50 (16.7)
45–54	134 (33.6)	100 (16.7)	66 (22.0)	50 (16.7)
55–64	146 (36.6)	100 (16.7)	104 (34.7)	50 (16.7)
≥65	38 (9.5)	100 (16.7)	76 (25.3)	50 (16.7)
Highest educational attainment				
Junior high school	0 (0.0)	8 (1.3)	0 (0.0)	5 (1.7)
High school	0 (0.0)	177 (29.5)	0 (0.0)	56 (18.7)
Vocational school/junior college	0 (0.0)	127 (21.2)	0 (0.0)	55 (18.3)
University	398 (99.7)	282 (47.0)	300 (100.0)	179 (59.7)
Other	1 (0.3)	6 (1.0)	0 (0.0)	5 (1.7)

In the 2018 survey, the reliability of the overall questionnaire was assessed using Cronbach’s alpha, confirming good internal consistency (α = 0.88). Using the same questionnaire, the 2025 survey yielded Cronbach’s alpha of 0.85; indicating maintained reliability.

Table 2 compares physicians responses from 2018 to 2025. Physicians’ mean score for “Intention to use” was 3.54 (SD = 1.02), not significantly different from 2018 (3.43; p = 0.16, d = 0.11). In contrast, perceived efficiency (“Do you think that using AI in healthcare would enable physicians to practice more efficiently?”) showed a significant increase, with a mean score of 3.62 (SD = 0.87) in 2025 compared with 3.44 (SD = 0.89) in 2018 (p < .01, d = 0.21).

**Table 2:** Comparison of the 2018 and 2025 survey results for physicians.

No.	Items	2018Mean(SD)	2025Mean(SD)	Cohen_d	95%CI	P value
1	Interest in topic	3.61(1.01)	3.59(1.06)	-0.01	(-0.16, 0.14)	0.87
2	Intention to use	3.43(1.00)	3.54(1.02)	0.11	(-0.04, 0.26)	0.16
3	Usefulness	3.72(0.93)	3.77(0.96)	0.05	(-0.10, 0.20)	0.48
4	User-friendliness	2.97(0.91)	3.01(0.97)	0.04	(-0.11, 0.19)	0.61
5	Necessity of legislation	4.01(1.01)	4.07(0.93)	0.07	(-0.08, 0.22)	0.35
6	Accuracy	3.11(0.84)	3.12(0.85)	0.01	(-0.14, 0.16)	0.91
7	Efficiency	3.44(0.89)	3.62(0.87)	0.21	(0.06, 0.36)	0.005
8	Relevance to life	3.62(0.90)	3.74(0.90)	0.14	(-0.01, 0.29)	0.07
9	Better medical services	3.73(0.85)	3.73(0.87)	0	(-0.15, 0.15)	0.99
10	Expectations of others	3.56(0.88)	3.59(0.91)	0.03	(-0.12, 0.18)	0.68
11	Expectations among patients	3.50(0.89)	3.50(0.88)	0	(-0.15, 0.15)	0.98
12	General impression	3.69(0.85)	3.70(0.88)	0.01	(-0.14, 0.16)	0.87
13	Mastery	3.15(0.95)	3.07(0.97)	-0.09	(-0.24, 0.06)	0.26
14	Necessity in medicine	3.74(0.84)	3.81(0.88)	0.08	(-0.07, 0.23)	0.31
15	Knowledge of AI in other contexts	2.88(1.00)	2.81(0.95)	-0.07	(-0.22, 0.08)	0.33
16	Knowledge of AI in medicine	2.66(1.00)	2.73(0.99)	0.07	(-0.08, 0.22)	0.34
17	Concern about accountability	3.91(1.01)	3.83(0.97)	-0.08	(-0.23, 0.07)	0.28
18	Concern about data leakage	3.38(1.08)	3.36(1.07)	-0.02	(-0.17, 0.13)	0.83
19	Brand impact	3.04(0.98)	2.95(1.03)	-0.09	(-0.24, 0.06)	0.23
20	Medical costs	3.01(0.94)	3.13(0.94)	0.13	(-0.02, 0.27)	0.1

Because the age distribution differed between the 2018 and 2025 physician samples, additional ordinal logistic regression analyses were conducted with adjustment for age and sex. After adjustment, survey year was not significantly associated with responses to either the item assessing intention to use medical AI (likelihood-ratio χ² = 0.84, p = 0.360) or the item assessing the potential of medical AI to improve clinical efficiency (likelihood-ratio χ² = 0.66, p = 0.415; Supplementary S1 Table). Age was significantly associated with both outcomes, whereas sex was not.

Table 3 compares survey results for the general public. Among members of the general public, the mean score for “intention to use” was 3.35 (SD = 0.90), showing no significant difference from the 2018 score (3.23; SD = 0.92) (p = .06, d = 0.13). In contrast, “knowledge of AI in medicine” (“I am well informed about the use of medical AI”) showed a significant increase, with a mean score of 2.29 (SD = 1.03) compared with 2.12 (SD = 1.01) in 2018 (p < .05, d = 0.16). In addition, the item assessing perceived impact of medical costs (“How do you think medical AI will affect medical costs? [1 = decrease, 5 = increase]”) had a mean score of 2.95 (SD = 0.96), representing a significant increase from the 2018 value of 2.79 (SD = 1.04) (p < .05, d = 0.17).

**Table 3:** Comparison of the 2018 and 2025 survey results for public.

No.	Items	2018Mean(SD)	2025Mean(SD)	Cohen_d	95%CI	P value
1	Interest in topic	3.46(0.99)	3.45(0.93)	-0.01	(-0.15, 0.13)	0.9
2	Intention to use	3.23(0.92)	3.35(0.90)	0.13	(-0.01, 0.27)	0.06
3	Usefulness	3.62(0.90)	3.56(0.96)	-0.07	(-0.20, 0.07)	0.37
4	User-friendliness	2.97(0.90)	2.97(0.94)	0	(-0.14, 0.14)	0.98
5	Necessity of legislation	3.98(0.97)	3.90(1.00)	-0.09	(-0.23, 0.05)	0.21
6	Accuracy	3.06(0.79)	3.12(0.77)	0.08	(-0.06, 0.22)	0.25
7	Efficiency	3.50(0.86)	3.54(0.79)	0.05	(-0.09, 0.19)	0.47
8	Relevance to life	3.66(0.92)	3.57(0.83)	-0.1	(-0.24, 0.04)	0.14
9	Better medical services	3.60(0.91)	3.57(0.81)	-0.04	(-0.18, 0.09)	0.52
10	Expectations of others	3.37(0.92)	3.34(0.89)	-0.03	(-0.17, 0.11)	0.66
11	Expectations among patients	3.46(0.94)	3.40(0.87)	-0.07	(-0.21, 0.07)	0.33
12	General impression	3.60(0.89)	3.52(0.85)	-0.09	(-0.23, 0.05)	0.19
13	Mastery	2.93(0.97)	3.01(0.88)	0.08	(-0.06, 0.22)	0.23
14	Necessity in medicine	3.67(0.97)	3.65(0.85)	-0.02	(-0.16, 0.12)	0.73
15	Knowledge of AI in other contexts	2.78(1.11)	2.83(1.11)	0.04	(-0.10, 0.18)	0.54
16	Knowledge of AI in medicine	2.12(1.01)	2.29(1.03)	0.17	(0.03, 0.31)	0.02
17	Concern about accountability	3.93(0.99)	3.83(0.96)	-0.11	(-0.24, 0.03)	0.13
18	Concern about data leakage	3.24(1.11)	3.22(1.01)	-0.02	(-0.15, 0.12)	0.82
19	Brand impact	3.06(0.99)	3.14(0.92)	0.09	(-0.05, 0.23)	0.2
20	Medical costs	2.79(1.04)	2.95(0.96)	0.16	(0.02, 0.30)	0.02

Table 4 presents the results of the exploratory factor analysis (EFA) using the 2018 dataset, indicating acceptance of medical AI classification into five factors: “Social Impression,” “Personal Acceptance,” “Knowledge,” “Risk and Accountability (Concerns),” and “Operational Quality.”

**Table 4:** Results of EFA.

No.	Questions	F1:	F2:	F3:	F4:	F5:	Factors Name
10	Expectations of others	0.816	0.2	0.09	0.026	0.233	F1
12	General impression	0.8	0.351	0.057	-0.032	0.093	
11	Expectations among patients	0.781	0.199	0.076	0.032	0.213	
14	Necessity in medicine	0.761	0.413	0.062	-0.008	0.058	
9	Better medical services	0.753	0.337	0.11	-0.058	0.083	
1	Interest in topic	0.446	0.343	0.25	-0.011	0.096	F2
8	Relevance to life	0.377	0.736	0.062	0.03	0.191	
3	Usefulness	0.447	0.7	0.111	-0.005	0.155	
7	Efficiency	0.32	0.679	0.065	0.002	0.311	
2	Intention to use	0.425	0.566	0.164	-0.065	0.298	
5	Necessity of legislation	0.19	0.517	0.018	0.326	-0.022	
20	Medical costs	-0.102	-0.183	-0.023	0.069	-0.073	
16	Knowledge of AI in medicine	0.082	0.002	0.822	-0.063	0.141	F3
15	Knowledge of AI in other contexts	0.12	0.139	0.699	0.041	0.006	
17	Concern about accountability	-0.044	0.095	-0.084	0.827	-0.18	F4
18	Concern about data leakage	-0.09	-0.147	-0.017	0.64	-0.061	
19	Brand impact	0.091	0.093	0.12	0.22	0.144	F5
4	User-friendliness	0.109	0.315	0.058	-0.081	0.581	
6	Accuracy	0.224	0.423	0.093	-0.063	0.515	
13	Mastery	0.415	0.001	0.1	-0.13	0.444	

Using the 2025 dataset (n = 600), confirmatory factor analysis (CFA) was conducted to evaluate this factor structure. Estimation was performed using the diagonally weighted least squares (DWLS) method, which is appropriate for ordinal data.

Table 5 summarizes the model fit indices for the five-factor and three-factor models. For the five-factor model evaluated using the 2025 dataset, the CFI, TLI, and SRMR were 0.988, 0.986, and 0.070, respectively. However, the RMSEA was 0.088 (90% CI, 0.083–0.094), and the robust RMSEA was 0.103, indicating limitations in overall model fit. For the three-factor model, the CFI and TLI exceeded 0.97 in both datasets. However, the RMSEA values were 0.121 (90% CI, 0.117–0.125) in 2018 and 0.106 (90% CI, 0.101–0.112) in 2025. Thus, despite the favorable CFI and TLI values, the three-factor model showed poor fit according to the RMSEA in both datasets.

**Table 5:** Model fit indices for the five-factor and three-factor confirmatory factor analysis models in the 2018 and 2025 datasets.

Model	Dataset	CFI	TLI	SRMR	RMSEA(90% CI)	Robust RMSEA
Five-factor model	2025	0.988	0.986	0.07	0.088 (0.083–0.094)	0.103
Three-factor model	2018	0.98	0.977	0.08	0.121 (0.117–0.125)	0.139
Three-factor model	2025	0.982	0.979	0.081	0.106 (0.101–0.112)	0.117

Table 6 presents standardized factor loadings for each factor. Many items in F1 (institutional and social evaluation) and F2 (personal acceptance) showed high factor loadings (generally ≥ 0.70). By contrast, Q5 and Q20 within F2 showed low factor loadings (Q5: 0.35; Q20: −0.117), suggesting weak correspondence.

**Table 6:** Standardized effect size from CFA.

	F1					F2						
	Q10	Q12	Q11	Q14	Q9	Q1	Q8	Q3	Q7	Q2	Q5	Q20
Standardized effect size	0.807	0.916	0.748	0.888	0.863	0.731	0.867	0.887	0.797	0.856	0.35	-0.117
	F3			F4		F5						
Standardized effect size	Q16	Q15	Q17	Q18	Q19	Q4	Q6	Q13				
	0.734	0.814	0.712	0.778	0.289	0.62	0.746	0.607				

Table 7 shows the correlations among factors. A very high correlation existed between F1 and F2 (r = 0.92). Relatively high positive correlations were also observed between F1 and F5 (performance and quality) and between F2 and F5 (r = 0.65–0.76), indicating limited independence among the five factors.

**Table 7:** Factor correlation from CFA.

	Covariance		Covariance		Covariance		Covariance
F1-F2	0.921	F2-F3	0.379	F3-F4	-0.22	F4-F5	-0.464
F1-F3	0.26	F2-F4	-0.178	F3-F5	0.383		
F1-F4	-0.168	F2-F5	0.755				
F1-F5	0.654						

Taken together, these results indicate that although the five-factor model has explanatory power, it also shows high inter-factor correlations and low loadings for some items.

Accordingly, to improve interpretability and explore a more parsimonious representation, we examined a three-factor model in which F1, F2, and F5 were combined into F′1 (overall acceptance), while F3 and F4 were retained as F′2 (knowledge) and F′3 (concerns), respectively. Using this three-factor structure, confirmatory factor analysis (CFA) was conducted on both the 2018 and 2025 datasets, and model fit indices and inter-factor correlations were calculated separately for each survey year.

Table 8 presents the standardized effect sizes (factor loadings) from the CFA based on the three-factor model. Both F′2 (knowledge) and F′3 (concerns) showed high standardized loadings and were structurally well defined, whereas F′1 included more heterogeneous acceptance-related items. These results indicate that, in medical AI acceptance, knowledge and concerns function as relatively independent and coherent constructs. Both F′2 (knowledge) and F′3 (concerns) showed high factor loadings and were structurally well defined. In medical AI acceptance, knowledge and concerns function as relatively independent constructs.

**Table 8:** Standardized effect size from CFA (3 Factors).

	F’1											
	Q10	Q12	Q11	Q14	Q9	Q1	Q8	Q3	Q7	Q2	Q5	Q20
2025	0.789	0.893	0.728	0.864	0.844	0.716	0.849	0.869	0.784	0.841	0.343	-0.115
2018	0.866	0.902	0.835	0.892	0.857	0.648	0.82	-0.865	0.783	0.816	0.383	-0.231
	F’1				F’2		F’3					
	Q19	Q4	Q6	Q13	Q16	Q15	Q17	Q18				
2025	0.253	0.48	0.579	0.466	0.728	0.821	0.608	0.911				
2018	0.208	0.478	0.613	0.482	0.732	0.912	0.436	1.395				

Table 9 presents correlations among three factors. A moderate positive correlation was observed between F′1 and F′2, and weak negative correlations were observed between F′1 and F′3, and between F′2 and F′3.

**Table 9:** Factor correlation from CFA (3 Factors).

Covariance	2025	2018
F’1-F’2	0.348	0.324
F’1-F’3	-0.219	-0.143
F’2-F’3	-0.207	-0.069

## Discussion

This repeated cross-sectional study compared attitudes toward relatively autonomous medical AI among physicians and members of the general public in Japan in 2018 and 2025. The main findings were that mean responses to the item assessing intention to use medical AI did not differ significantly between 2018 and 2025 in either group. In contrast, physicians showed higher mean responses to the item concerning the potential of medical AI to improve clinical efficiency, while members of the general public showed higher mean responses to items assessing self-rated knowledge and the possibility that medical AI could increase healthcare costs. These findings suggest that greater familiarity with and recognition of the practical implications of medical AI did not translate directly into greater willingness to use it. The factor analyses further suggested that attitudes toward medical AI are multidimensional, encompassing overall acceptance, knowledge, and concerns; however, limitations in model fit indicate that the proposed factor structure should be interpreted cautiously. Overall, the results suggest that acceptance of relatively autonomous medical AI reflects a complex balance between perceived usefulness, increasing knowledge, and persistent concerns rather than a simple increase over time.

Among physicians, mean responses to the item assessing intention to use medical AI did not differ significantly between 2018 and 2025. In the unadjusted comparison, physicians surveyed in 2025 showed higher mean responses to the item assessing the potential of medical AI to improve clinical efficiency. However, this difference was no longer statistically significant after adjustment for age and sex, suggesting that differences in the age composition of the two physician samples may have contributed to the observed unadjusted difference. Thus, the present findings do not provide clear evidence that physicians’ perceptions of the efficiency benefits of medical AI increased independently of differences in sample characteristics.

Several factors may explain why higher familiarity with medical AI did not translate into a significant increase in intention to use it. First, the questionnaire described a relatively autonomous form of medical AI that determines diagnoses and treatment plans without physician involvement. Greater exposure to general-purpose AI or physician-assistive systems may therefore not lead to greater willingness to use the more autonomous system described in this study. Second, increased knowledge may heighten awareness not only of potential benefits but also of issues related to safety, diagnostic responsibility, accountability, medical ethics, and legal regulation [17]. Among members of the general public, the higher rating concerning possible increases in healthcare costs may also have restrained willingness to use medical AI. These considerations suggest that intention to use medical AI may depend on whether perceived benefits outweigh concerns regarding autonomy, risk, responsibility, and cost, rather than on familiarity or knowledge alone [18]. However, because the questionnaire did not directly assess participants’ reasons for their intention to use or not use medical AI, these explanations should be regarded as hypotheses rather than conclusions demonstrated by the present data.

Among members of the general public, intention to use medical AI did not show a statistically significant change from 2018 to 2025, although the mean score increased slightly. By contrast, self-rated knowledge of medical AI (“I am well informed about the use of medical AI”) increased significantly, suggesting that self-perceived knowledge of medical AI has increased. Furthermore, perceptions that medical AI would lead to increased healthcare costs also increased significantly. Taken together, these results suggest that among members of the general public, knowledge of medical AI has increased, while awareness of its social and economic implications has deepened simultaneously. In this regard, Richardson et al. reported that although patients and the general public express positive interest in medical AI, they simultaneously harbor concerns regarding **safety, accountability, and healthcare costs**, indicating that increased understanding of AI **does not necessarily translate into a straightforward increase in intentio**n to use it (Richardson et al., 2021) [19]. Medical AI thus appears to be transitioning from a stage in which it is perceived merely as a novel and promising technology to one in which it is evaluated more realistically, including in relation to healthcare systems and costs, which may restrain a rapid rise in intention to use. It should also be noted that perceptions of the impact of medical AI on healthcare costs vary by country and healthcare system; for example, expectations regarding cost containment have been reported in the United States (Nong et al., 2025) [20].

The definition of medical AI used in this study should also be considered when interpreting these findings. The questionnaire described a relatively autonomous form of medical AI in which AI determines diagnoses and treatment plans without requiring physician involvement. This wording, which was originally used in the 2018 survey, was retained unchanged in the 2025 survey to maintain measurement consistency and enable direct comparison between the two survey time points. However, this definition is narrower than the broader range of general-purpose health-related AI applications and AI-assisted clinical decision-support systems to which physicians and the general public may have been increasingly exposed between 2018 and 2025. Therefore, the observed increase in self-rated knowledge or exposure to information about AI may not directly indicate greater familiarity with or acceptance of the autonomous form of medical AI described in the questionnaire. The present findings should thus be interpreted as reflecting attitudes toward this specific form of medical AI rather than toward medical AI in general.

The EFA using the 2018 dataset suggested that attitudes toward medical AI may be represented by five factors: social impression, personal acceptance, knowledge, concerns, and operational quality. However, CFA using the 2025 dataset indicated limited discriminant validity of the five-factor model. In particular, the correlation between F1 (social impression) and F2 (personal acceptance) was very high (r = 0.92), while F5 (operational quality) was also substantially correlated with F1 (r = 0.65) and F2 (r = 0.76). Conceptually, these three factors comprised closely related positive evaluations of medical AI, including favorable social perceptions, personal willingness to accept or use it, and evaluations of its usefulness and operational performance. We therefore explored a more parsimonious three-factor structure by combining F1, F2, and F5 into a broader factor termed “overall acceptance of medical AI,” while retaining knowledge and concerns as separate factors. However, the simplified model did not demonstrate satisfactory overall fit. Although the CFI and TLI values were favorable, the RMSEA exceeded 0.10 in both datasets. Therefore, the three-factor structure should be regarded as exploratory and requires further validation.

The exploratory analyses suggested that overall acceptance, knowledge, and concerns may represent broad dimensions of attitudes toward medical AI. Although broadly similar dimensions emerged in the two datasets, the equivalence of the measurement structure across survey years could not be established because formal measurement-invariance testing was not conducted. Within the exploratory three-factor model, knowledge and concerns were represented as separate dimensions and showed associations with overall acceptance.

This structure is also consistent with findings by Shevtsova et al. (2024), **who conceptualized trust in and acceptance of medical** AI **as multidimensional constructs shaped by both knowledge-related aspects, such as comprehensibility and transparency of the technology, and concerns related to ethical and legal issues [**21**].** A weak negative correlation between knowledge and concerns suggests that increases in knowledge do not necessarily reduce concerns. Moreover, the finding that attitudes toward medical AI comprise multiple dimensions, including positive evaluations as well as concerns and cautious stances, is consistent with Heinrichs et al. (2025), who conducted exploratory factor analysis among physicians and identified relatively independent factors of “acceptance and expectations toward AI” and “skepticism and anxiety toward AI” [22]. In other words, acceptance of medical AI may not lie on a single continuum, but instead exhibit a multidimensional structure in which positive evaluations and concerns coexist.

These results indicate that acceptance of medical AI does not increase unidirectionally through deeper technical understanding alone, but rather represents a complex process in which increased knowledge may coexist with persistent risk perceptions and concerns regarding accountability. Although broadly similar dimensions emerged in the 2018 and 2025 datasets, the stability or equivalence of the underlying measurement structure across survey years could not be established because formal measurement-invariance testing was not conducted. The coexistence of greater knowledge and persistent concerns may have important implications for the future implementation of medical AI.

## Limitations

This study has several limitations.

First, this study was based on repeated cross-sectional surveys conducted in 2018 and 2025 and did not follow the same individuals over time. Therefore, the relationships observed among knowledge, concerns, and acceptance of medical AI were correlational and do not demonstrate causality. Nevertheless, the seven-year interval provided an opportunity to compare item-level response patterns during a period of substantial development and increasing societal visibility of medical AI.

Second, no formal a priori sample size calculation was conducted, and the 2025 sample size was determined based on budgetary and feasibility considerations.

Third, formal measurement-invariance testing across the 2018 and 2025 surveys was not conducted. Accordingly, configural, metric, and scalar invariance were not established, and the equivalence of the questionnaire’s measurement properties across survey years cannot be assumed. The comparisons in this study were based on observed responses to individual questionnaire items rather than latent factor means or composite scores. Therefore, the findings should be interpreted as item-level comparisons and not as definitive evidence of changes or stability in latent acceptance constructs over time.

Fourth, the simplified three-factor model did not demonstrate consistently satisfactory fit across the evaluated indices. In particular, the RMSEA exceeded 0.10 in both survey years, indicating poor model fit. Accordingly, the proposed three-factor structure should be considered exploratory rather than a validated measurement model. Further studies should refine the questionnaire based on a clearer theoretical framework and validate the revised structure in independent samples.

Fifth, the questionnaire used a relatively restrictive definition of medical AI that emphasized autonomous diagnosis and treatment planning without physician involvement. Although the same wording was retained in 2025 to ensure comparability with the 2018 survey, this definition may not reflect the broader range of general-purpose health-related AI applications and physician-assistive decision-support systems that participants may have encountered. Therefore, the findings may not be generalizable to attitudes toward medical AI in general, particularly less autonomous or physician-supervised forms of AI.

Sixth, the questionnaire did not directly assess the reasons underlying participants’ intention to use or not use medical AI. Therefore, the explanations proposed for the absence of a significant increase in intention to use are interpretive and should be examined directly in future studies using measures of perceived risk, trust, responsibility, autonomy, and cost concerns.

Finally, this study was conducted within the context of Japan’s healthcare system and sociocultural context, which limits generalizability. However, because Japan is an aging society where the introduction of medical AI has progressed relatively early, the findings of this study may nonetheless provide informative insights for other countries in which the societal implementation of medical AI is advancing.

## Conclusions

Using repeated cross-sectional online survey data collected in Japan in 2018 and 2025, this study found that intention to use relatively autonomous medical AI did not differ significantly between survey years among either physicians or members of the general public. Among physicians, the unadjusted increase in perceived clinical efficiency was not significant after adjustment for age and sex. Among members of the general public, self-rated knowledge was higher in 2025, while expectations that medical AI would increase healthcare costs were also greater. These findings suggest that greater familiarity or knowledge does not necessarily lead to greater willingness to use medical AI. Promoting informed acceptance will require addressing persistent concerns alongside efforts to improve understanding.

## Supporting information

S1 Table Age- and sex-adjusted ordinal logistic regression analyses of physicians’ responses. (DOCX)

S2 Table Distribution of self-reported medical specialties among physicians in the 2018 and 2025 surveys. (DOCX)
