# Supplementary material for: Changes in the acceptance of artificial intelligence in medicine among physicians and the general public in Japan: A repeated cross-sectional comparison of 2018 and 2025
Source: PLOS Digit Health. 2026 Oct 23;5(10):e0001769. doi: 10.1371/journal.pdig.0001769 (PMC13693883; doi:10.1371/journal.pdig.0001769)
Supplement: Supplementary file 1 [file pdig.0001769.s001.docx]

S1 Table. Age- and sex-adjusted ordinal logistic regression analyses of physicians’ responses

| **Outcome** | **Predictor** | **Likelihood-ratio χ²** | **p value** |
| --- | --- | --- | --- |
| Intention to use medical AI | Survey year | 0.84 | 0.36 |
|  | Age | 12.08 | <0.001 |
|  | Sex | 0.94 | 0.333 |
| Perceived improvement in clinical efficiency | Survey year | 0.66 | 0.415 |
|  | Age | 11.38 | <0.001 |
|  | Sex | 0.46 | 0.498 |

**Note:** Separate ordinal logistic regression models were fitted for each outcome among physicians. Survey year was the primary explanatory variable, with age and sex included as covariates.
