# Supplementary material for: Changes in the acceptance of artificial intelligence in medicine among physicians and the general public in Japan: A repeated cross-sectional comparison of 2018 and 2025
Source: PLOS Digit Health. 2026 Oct 23;5(10):e0001769. doi: 10.1371/journal.pdig.0001769 (PMC13693883; doi:10.1371/journal.pdig.0001769)
Supplement: Supplementary file 2 [file pdig.0001769.s002.docx]

S2 Table. Distribution of self-reported medical specialties among physicians in the 2018 and 2025 surveys

| **Medical specialty** | **2018 physicians** | **(%)** | **2025 physicians** | **(%)** |
| --- | --- | --- | --- | --- |
| Internal Medicine | 101 | 25.3 | 77 | 25.7 |
| Psychiatry | 21 | 5.3 | 12 | 4.0 |
| Neurology | 6 | 1.5 | 5 | 1.7 |
| Respiratory Medicine | 11 | 2.8 | 8 | 2.7 |
| Gastroenterology | 20 | 5.0 | 13 | 4.3 |
| Cardiology | 11 | 2.8 | 15 | 5.0 |
| Pediatrics | 20 | 5.0 | 16 | 5.3 |
| Surgery | 25 | 6.3 | 29 | 9.7 |
| Orthopedic Surgery | 37 | 9.3 | 16 | 5.3 |
| Plastic and Reconstructive Surgery | 4 | 1.0 | 3 | 1.0 |
| Cosmetic Surgery | 0 | 0.0 | 3 | 1.0 |
| Neurosurgery | 12 | 3.0 | 11 | 3.7 |
| Thoracic Surgery | 5 | 1.3 | 3 | 1.0 |
| Cardiovascular Surgery | 6 | 1.5 | 3 | 1.0 |
| Pediatric Surgery | 1 | 0.3 | 0 | 0.0 |
| Dermatology | 10 | 2.5 | 2 | 0.7 |
| Urology | 5 | 1.3 | 10 | 3.3 |
| Venereology | 1 | 0.3 | 0 | 0.0 |
| Proctology | 0 | 0.0 | 1 | 0.3 |
| Obstetrics and Gynecology | 17 | 4.3 | 7 | 2.3 |
| Gynecology | 0 | 0.0 | 3 | 1.0 |
| Ophthalmology | 12 | 3.0 | 13 | 4.3 |
| Otorhinolaryngology | 9 | 2.3 | 7 | 2.3 |
| Radiology | 12 | 3.0 | 6 | 2.0 |
| Anesthesiology | 20 | 5.0 | 12 | 4.0 |
| Rheumatology | 1 | 0.3 | 0 | 0.0 |
| Rehabilitation Medicine | 7 | 1.8 | 5 | 1.7 |
| Other | 25 | 6.3 | 20 | 6.7 |
| Total | 399 | 100.0 | 300 | 100.0 |

**Note:** Medical specialty was self-reported. Percentages were calculated using the number of physicians included in the final analytical sample in each survey year as the denominator (2018, n = 399; 2025, n = 300).
