# Supplementary material for: Changes in the acceptance of artificial intelligence in medicine among physicians and the general public in Japan: A repeated cross-sectional comparison of 2018 and 2025
Source: PLOS Digit Health. 2026 Oct 23;5(10):e0001769. doi: 10.1371/journal.pdig.0001769 (PMC13693883; doi:10.1371/journal.pdig.0001769)
Supplement: Supplementary file 3 [file pdig.0001769.s004.docx]

Dear Reviewer [1] ,

We would like to thank you for your thoughtful and constructive comments. We have carefully revised the manuscript to address all the issues you raised. Below, we provide point-by-point responses to your comments, with explanations of how we have addressed each one in the revised manuscript.

---

Reviewer #1:

Comment 1

 - Introduction
- This is a study addressing a highly relevant question regarding the acceptance and attitudes towards the use of medical AI among physicians and the general public. The relevance of this question and the need for the study are well-articulated.
- I don’t think this study should be called a “seven-year comparative study (2018-2025)”, which sounds like a repeated cross-sectional study conducted every year for the seven years. Rather, it is a repeated cross-sectional study comparing 2018 and 2025. In a similar vein, I wouldn’t call it a trend study because only two time points are compared.

Response:

Thank you for your valuable comment. In response to your suggestion, we have revised the title as follows:

“Changes in the Acceptance of Artificial Intelligence in Medicine Among Physicians and the General Public in Japan: A Repeated Cross-Sectional Comparison of 2018 and 2025”

Comment 2
- Methods
- The definition of medical AI is quite restrictive, and this nuance and its potential impact on interpretation of the results should be mentioned in the Discussion. “Healthcare in which AI determines diagnoses and treatment plans without requiring physicians” are high-risk devices that require stringent regulatory approvals. In contrast, the increase in the exposure and knowledge regarding AI is likely to have been more on the general purpose health-related AI or AI-based assistive devices for physicians’ decision making. This nuance/discrepancy should be taken into account when interpreting the results and considering the implications of the results.

Response:

Thank you for this important comment. We agree that the definition of medical AI used in the questionnaire was relatively restrictive and focused on a highly autonomous form of AI. The wording used in the 2018 survey was retained unchanged in 2025 to ensure comparability between the two survey time points.

We have revised the Discussion to clarify that increased exposure to general-purpose health-related AI or AI-assisted clinical decision-support systems may not directly translate into increased acceptance of autonomous medical AI. We have also added this issue as a limitation and clarified that the findings should be interpreted as reflecting attitudes toward the specific form of medical AI described in the questionnaire, rather than toward medical AI in general. These revisions appear in the Discussion (Lines 358-369) and Limitations sections (Lines 434–440).

Discussion

The definition of medical AI used in this study should also be considered when interpreting these findings. The questionnaire described a relatively autonomous form of medical AI in which AI determines diagnoses and treatment plans without requiring physician involvement. This wording, which was originally used in the 2018 survey, was retained unchanged in the 2025 survey to maintain measurement consistency and enable direct comparison between the two survey time points. However, this definition is narrower than the broader range of general-purpose health-related AI applications and AI-assisted clinical decision-support systems to which physicians and the general public may have been increasingly exposed between 2018 and 2025. Therefore, the observed increase in self-rated knowledge or exposure to information about AI may not directly indicate greater familiarity with or acceptance of the autonomous form of medical AI described in the questionnaire. The present findings should thus be interpreted as reflecting attitudes toward this specific form of medical AI rather than toward medical AI in general.

Limitations

Fifth, the questionnaire used a relatively restrictive definition of medical AI that emphasized autonomous diagnosis and treatment planning without physician involvement. Although the same wording was retained in 2025 to ensure comparability with the 2018 survey, this definition may not reflect the broader range of general-purpose health-related AI applications and physician-assistive decision-support systems that participants may have encountered. Therefore, the findings may not be generalizable to attitudes toward medical AI in general, particularly less autonomous or physician-supervised forms of AI.

Comment 3

- Please report the sampling/recruitment method. I would like to see more details than the fact that it was conducted online by a company called Rakuten Insight. Methodological details may matter for the interpretation of the results.

Response：

Thank you for this important comment. We agree that the original manuscript did not provide sufficient details regarding the sampling and recruitment procedures.

We have expanded the Methods section to clarify that participants in both surveys were recruited from a commercial opt-in online research panel maintained by Rakuten Insight, Inc., Japan. We have also added details regarding the composition of the samples and the classification of physicians and members of the general public.

In the 2018 survey, the general public sample consisted of 600 participants across six age groups, with 50 men and 50 women in each age group. The physician sample initially consisted of 400 participants aged 25 years or older, comprising 350 men and 50 women. One respondent was excluded because the reported educational attainment was inconsistent with the educational requirements for obtaining a medical license in Japan, resulting in a final analytical sample of 399 physicians. In the 2025 survey, the sample consisted of 600 participants, comprising 300 physicians and 300 members of the general public.

We have further clarified that physician status was determined based on participants’ self-reported occupation and was not independently verified. These revisions appear in the Methods section (Lines 127–144).

**Participants and sample selection**

Participants in both surveys were recruited from a commercial opt-in online research panel maintained by Rakuten Insight, Inc., Japan. Physician status was determined based on participants’ self-reported occupation and was not independently verified. Physician participants also self-reported their medical specialty by selecting one specialty category.

The 2018 survey initially included 600 members of the general public and 400 self-identified physicians. The general public sample was divided into six age groups (15–24, 25–34, 35–44, 45–54, 55–64, and ≥65 years), with 50 men and 50 women in each group. The physician sample consisted of participants aged ≥25 years and included 350 men and 50 women. Physicians were recruited regardless of clinical experience or medical specialty. One respondent who self-identified as a physician was excluded because the reported highest educational attainment was vocational school/junior college, which was inconsistent with the educational requirements for obtaining a medical license in Japan. The final 2018 analytical sample therefore consisted of 399 physicians and 600 members of the general public.

The 2025 survey included 600 participants, comprising 300 self-identified physicians and 300 members of the general public. No formal a priori sample size calculation was conducted. The target sample size of 300 physicians and 300 members of the general public was determined based on the available research budget and the feasibility of recruitment through the commercial online panel.

Comment 4
- Results
- Table 1 and 2 can be combined. The combined table can retain the format of the current Table 2, and the rows for sex and age group can be added. Please report percentages so that is is easier to compare across doctor vs. public and across 2018 vs. 2025.

Response：

Thank you for this helpful suggestion. We have combined the former Tables 1 and 2 into a single table and added rows for sex and age group. All values are now reported as n (%) to facilitate comparisons between physicians and members of the public and between the 2018 and 2025 samples. The revised table is presented as Table 1.

Comment 5
- Table 3 in the current form is not very informative because there is no meaningful target population that the sample (physician+public) represents. Instead, it might be more informative to have Table 3 to describe the difference between the physicians vs. public in 2018, and the difference between the physicians vs. public in 2025. These are only suggestions, and if these are not the authors’ questions, simply Table 3 can be deleted.

Response:

Thank you for this helpful comment. We agree that the combined sample of physicians and members of the general public does not represent a clearly defined target population. Because our primary objective was to compare the 2018 and 2025 responses separately among physicians and members of the general public, we deleted the former Table 3 and removed the corresponding statements based on the combined sample. We also revised the opening section of the Discussion to summarize and interpret the findings separately for physicians and members of the general public. The subsequent tables and all corresponding references in the text have been renumbered accordingly. These revisions appear on pages 14–15, lines 304–339.

Discussion:

This repeated cross-sectional study compared attitudes toward relatively autonomous medical AI among physicians and members of the general public in Japan in 2018 and 2025. The main findings were that mean responses to the item assessing intention to use medical AI did not differ significantly between 2018 and 2025 in either group. In contrast, physicians showed higher mean responses to the item concerning the potential of medical AI to improve clinical efficiency, while members of the general public showed higher mean responses to items assessing self-rated knowledge and the possibility that medical AI could increase healthcare costs. These findings suggest that greater familiarity with and recognition of the practical implications of medical AI did not translate directly into greater willingness to use it. The factor analyses further suggested that attitudes toward medical AI are multidimensional, encompassing overall acceptance, knowledge, and concerns; however, limitations in model fit indicate that the proposed factor structure should be interpreted cautiously. Overall, the results suggest that acceptance of relatively autonomous medical AI reflects a complex balance between perceived usefulness, increasing knowledge, and persistent concerns rather than a simple increase over time.

Among physicians, mean responses to the item assessing intention to use medical AI did not differ significantly between 2018 and 2025. In the unadjusted comparison, physicians surveyed in 2025 showed higher mean responses to the item assessing the potential of medical AI to improve clinical efficiency. However, this difference was no longer statistically significant after adjustment for age and sex, suggesting that differences in the age composition of the two physician samples may have contributed to the observed unadjusted difference. Thus, the present findings do not provide clear evidence that physicians’ perceptions of the efficiency benefits of medical AI increased independently of differences in sample characteristics.

Several factors may explain why higher familiarity with medical AI did not translate into a significant increase in intention to use it. First, the questionnaire described a relatively autonomous form of medical AI that determines diagnoses and treatment plans without physician involvement. Greater exposure to general-purpose AI or physician-assistive systems may therefore not lead to greater willingness to use the more autonomous system described in this study. Second, increased knowledge may heighten awareness not only of potential benefits but also of issues related to safety, diagnostic responsibility, accountability, medical ethics, and legal regulation. Among members of the general public, the higher rating concerning possible increases in healthcare costs may also have restrained willingness to use medical AI. These considerations suggest that intention to use medical AI may depend on whether perceived benefits outweigh concerns regarding autonomy, risk, responsibility, and cost, rather than on familiarity or knowledge alone. However, because the questionnaire did not directly assess participants’ reasons for their intention to use or not use medical AI, these explanations should be regarded as hypotheses rather than conclusions demonstrated by the present data.

Comment 6
- All acronyms should be defined (SRMR, TLI, RMSEA, etc.).

Response:

Thank you for pointing this out. We have defined all acronyms at their first occurrence in the manuscript, including exploratory factor analysis (EFA), confirmatory factor analysis (CFA), comparative fit index (CFI), Tucker–Lewis index (TLI), root mean square error of approximation (RMSEA), standardized root mean square residual (SRMR), and diagonally weighted least squares (DWLS).

Comment 7
- There are too many tables. More thought should be put into the presentations of the findings. For example, Table 7 and 10 can be combined. Some tables could be supplementary. There is no correct way but please try to improve the presentations.

Response:

Thank you for this helpful comment. We agree that the original presentation included too many separate tables and made the findings unnecessarily fragmented. To improve clarity, we combined the former Tables 7 and 10 into a single table presenting the model fit indices for the five-factor and three-factor CFA models across the 2018 and 2025 datasets. We also revised the corresponding CFA results paragraph to report these findings together and facilitate direct comparison across models and survey years. In addition, the subsequent table numbers and all corresponding references in the manuscript were updated accordingly. These revisions appear on page 11-12, lines 256–262, and in Table 5.

Table 5 summarizes the model fit indices for the five-factor and three-factor models. For the five-factor model evaluated using the 2025 dataset, the CFI, TLI, and SRMR were 0.988, 0.986, and 0.070, respectively. However, the RMSEA was 0.088 (90% CI, 0.083–0.094), and the robust RMSEA was 0.103, indicating limitations in overall model fit. For the three-factor model, the CFI and TLI exceeded 0.97 in both datasets. However, the RMSEA values were 0.121 (90% CI, 0.117–0.125) in 2018 and 0.106 (90% CI, 0.101–0.112) in 2025. Thus, despite the favorable CFI and TLI values, the three-factor model showed poor fit according to the RMSEA in both datasets.

Comment 8:
- Discussion
- Please use the first paragraph to summarize and interpret the main findings of the study.

Response:

Thank you for this helpful suggestion. We have revised the first paragraph of the Discussion to summarize and interpret the main findings of the study. The revised paragraph now highlights the differences in changes between physicians and members of the general public, the variation across questionnaire items, and the multidimensional nature of acceptance of relatively autonomous medical AI. We have also added a cautious interpretation of the factor-analytic findings in light of the limitations in model fit. Detailed interpretations of the findings for physicians, members of the general public, and the factor structure are presented in the subsequent paragraphs. These revisions appear in the Discussion section (Lines 304–317).

**Discussion**

This repeated cross-sectional study compared attitudes toward relatively autonomous medical AI among physicians and members of the general public in Japan in 2018 and 2025. The main findings were that mean responses to the item assessing intention to use medical AI did not differ significantly between 2018 and 2025 in either group. In contrast, physicians showed higher mean responses to the item concerning the potential of medical AI to improve clinical efficiency, while members of the general public showed higher mean responses to items assessing self-rated knowledge and the possibility that medical AI could increase healthcare costs. These findings suggest that greater familiarity with and recognition of the practical implications of medical AI did not translate directly into greater willingness to use it. The factor analyses further suggested that attitudes toward medical AI are multidimensional, encompassing overall acceptance, knowledge, and concerns; however, limitations in model fit indicate that the proposed factor structure should be interpreted cautiously. Overall, the results suggest that acceptance of relatively autonomous medical AI reflects a complex balance between perceived usefulness, increasing knowledge, and persistent concerns rather than a simple increase over time.

Comment9:
- There is no need to directly refer to the Results when discussing the results. You can delete phrases like “as shown in Table 4”.

Response:

Thank you for this comment. We have removed direct references to the Results tables from the Discussion, including phrases such as “As shown in Table 4” and “As shown in Table 5.” The Discussion now focuses on interpreting the findings rather than directing readers back to the Results section.

Please let us know if further clarification or revisions are needed.

Dear Reviewer [2] ,

We would like to thank you for your thoughtful and constructive comments. We have carefully revised the manuscript to address all the issues you raised. Below, we provide point-by-point responses to your comments, with explanations of how we have addressed each one in the revised manuscript.

----

Reviewer #2:

This study examines changes in medical AI acceptance among Japanese physicians and the public over 7 years (2018–2025) using identical questionnaires. The core finding—that perceived usefulness remained high but intention to use did not significantly increase, while concerns persisted—is relevant for AI implementation policy. The study is methodologically sound with clear presentation, but suffers from largely confirmatory findings, suboptimal factor model fit, and lack of measurement invariance testing across survey years.

Comment1:
The 5-factor EFA model (Table 6) was abandoned after CFA showed high inter-factor correlations (r=0.92 between F1-F2) and poor discriminant validity. The simplified 3-factor model still shows RMSEA >0.10 in both years (2025: 0.106; 2018: 0.121), well above the 0.08 cutoff. Authors dismiss this as "some conceptual heterogeneity" (line 299), but this indicates poor model fit.

Response:

Thank you for this important comment. We agree that our original wording understated the limitations of the three-factor model. Although the CFI and TLI values were favorable, the RMSEA exceeded 0.10 in both the 2018 and 2025 datasets, indicating poor model fit according to this index. We have therefore revised the Results and Discussion to state this limitation explicitly. We also removed the phrase “some conceptual heterogeneity” and revised statements suggesting acceptable fit or structural stability. The three-factor model is now described as an exploratory, more parsimonious representation rather than as a validated factor structure. These revisions appear on page 11-12, lines 256–262, and page 16, lines 371–383.

Results:

Table 5 summarizes the model fit indices for the five-factor and three-factor models. For the five-factor model evaluated using the 2025 dataset, the CFI, TLI, and SRMR were 0.988, 0.986, and 0.070, respectively. However, the RMSEA was 0.088 (90% CI, 0.083–0.094), and the robust RMSEA was 0.103, indicating limitations in overall model fit. For the three-factor model, the CFI and TLI exceeded 0.97 in both datasets. However, the RMSEA values were 0.121 (90% CI, 0.117–0.125) in 2018 and 0.106 (90% CI, 0.101–0.112) in 2025. Thus, despite the favorable CFI and TLI values, the three-factor model showed poor fit according to the RMSEA in both datasets.

Discussion:

The EFA using the 2018 dataset suggested that attitudes toward medical AI may be represented by five factors: social impression, personal acceptance, knowledge, concerns, and operational quality. However, CFA using the 2025 dataset indicated limited discriminant validity of the five-factor model. In particular, the correlation between F1 (social impression) and F2 (personal acceptance) was very high (r = 0.92), while F5 (operational quality) was also substantially correlated with F1 (r = 0.65) and F2 (r = 0.76). Conceptually, these three factors comprised closely related positive evaluations of medical AI, including favorable social perceptions, personal willingness to accept or use it, and evaluations of its usefulness and operational performance. We therefore explored a more parsimonious three-factor structure by combining F1, F2, and F5 into a broader factor termed “overall acceptance of medical AI,” while retaining knowledge and concerns as separate factors. However, the simplified model did not demonstrate satisfactory overall fit. Although the CFI and TLI values were favorable, the RMSEA exceeded 0.10 in both datasets. Therefore, the three-factor structure should be regarded as exploratory and requires further validation.

Comment 2:
Authors state: "measurement invariance was not examined stepwise" (line 413). Without establishing configural, metric, and scalar invariance, comparing mean scores across 2018 vs. 2025 is not justified. This is a fundamental requirement for longitudinal comparisons [Vandenberg & Lance, 2000].

Response:

Thank you for this important comment. We agree that measurement invariance is necessary for rigorous comparisons of latent constructs across survey years. However, the primary comparisons in this study were based on the observed mean responses to individual questionnaire items rather than on latent factor means or composite scale scores. Therefore, we did not compare latent factor means across survey years. Nevertheless, we acknowledge that the absence of formal tests of configural, metric, and scalar invariance limits our ability to conclude that the measurement properties of the questionnaire were equivalent across 2018 and 2025. We have therefore revised the manuscript to clarify that the findings represent item-level comparisons of observed responses rather than changes in latent acceptance constructs. We have also moderated statements implying temporal stability or change in the underlying factor structure and strengthened the corresponding limitation.

These revisions appear in the Methods on page 7, lines 191–193, in the Discussion on page 17, lines 406–408, and in the Limitations on page 17, lines 422–428.

Methods:

Comparisons between the 2018 and 2025 surveys were conducted at the level of individual questionnaire items. The study did not compare latent factor means or composite scale scores across survey years.

Discussion:

Although broadly similar dimensions emerged in the 2018 and 2025 datasets, the stability or equivalence of the underlying measurement structure across survey years could not be established because formal measurement-invariance testing was not conducted.

Limitations:

Third, formal measurement-invariance testing across the 2018 and 2025 surveys was not conducted. Accordingly, configural, metric, and scalar invariance were not established, and the equivalence of the questionnaire’s measurement properties across survey years cannot be assumed. The comparisons in this study were based on observed responses to individual questionnaire items rather than latent factor means or composite scores. Therefore, the findings should be interpreted as item-level comparisons and not as definitive evidence of changes or stability in latent acceptance constructs over time.

Comment3:
The rationale for collapsing F1, F2, F5 into F'1 is vague: "prioritized interpretability and structural stability" (line 288).

Response:

Thank you for this important comment. We agree that the rationale for combining F1, F2, and F5 was insufficiently explained in the original manuscript. We have revised the text to clarify both the statistical and conceptual basis for this exploratory simplification. Specifically, F1 (social impression) and F2 (personal acceptance) showed a very high inter-factor correlation (r = 0.92), indicating limited discriminant validity. F5 (operational quality) was also substantially correlated with F1 and F2, and its items reflected favorable evaluations of the usefulness and performance of medical AI. Because these three factors represented closely related positive evaluations of medical AI, we explored a broader factor termed “overall acceptance of medical AI.” We have also clarified that this three-factor structure was examined as an exploratory, more parsimonious alternative and should not be regarded as a validated factor structure, given its unsatisfactory RMSEA values. These revisions appear in the Discussion on page 16, lines 371–383.

Discussion:

The EFA using the 2018 dataset suggested that attitudes toward medical AI may be represented by five factors: social impression, personal acceptance, knowledge, concerns, and operational quality. However, CFA using the 2025 dataset indicated limited discriminant validity of the five-factor model. In particular, the correlation between F1 (social impression) and F2 (personal acceptance) was very high (r = 0.92), while F5 (operational quality) was also substantially correlated with F1 (r = 0.65) and F2 (r = 0.76). Conceptually, these three factors comprised closely related positive evaluations of medical AI, including favorable social perceptions, personal willingness to accept or use it, and evaluations of its usefulness and operational performance. We therefore explored a more parsimonious three-factor structure by combining F1, F2, and F5 into a broader factor termed “overall acceptance of medical AI,” while retaining knowledge and concerns as separate factors. However, the simplified model did not demonstrate satisfactory overall fit. Although the CFI and TLI values were favorable, the RMSEA exceeded 0.10 in both datasets. Therefore, the three-factor structure should be regarded as exploratory and requires further validation.

Comment4:
Age distribution differs markedly between years (Table 1): 2018 physicians skewed older (45-64), 2025 more balanced.

Response:

Thank you for highlighting this important difference in sample composition. We agree that the physician samples differed in age distribution between 2018 and 2025, with the 2018 sample including a larger proportion of physicians aged 45–64 years. To assess whether this difference influenced the principal findings, we conducted additional ordinal logistic regression analyses for the items assessing intention to use medical AI and the perceived potential of medical AI to improve clinical efficiency. Survey year was included as the primary explanatory variable, with age and sex included as covariates. After adjustment, survey year was not significantly associated with intention to use medical AI (likelihood-ratio χ² = 0.84, p = 0.360) or perceived improvement in clinical efficiency (likelihood-ratio χ² = 0.66, p = 0.415). These findings suggest that the unadjusted difference in perceived efficiency may have been partly attributable to differences in the age composition of the physician samples. We have added these analyses to the Methods and Results, presented the detailed findings in Supplementary Table S1, and revised the Discussion accordingly.

These revisions appear in the Methods on page 5, lines 163–167, in the Results on page 9, lines 231–236, in Supplementary Table S1, and in the Discussion on page 14, lines 318–325.

Methods:

Because the age distribution differed between the 2018 and 2025 physician samples, additional ordinal logistic regression analyses were conducted as sensitivity analyses. Separate models were fitted for the items assessing intention to use medical AI and the perceived potential of medical AI to improve clinical efficiency. Survey year was included as the primary explanatory variable, with age and sex included as covariates. The questionnaire responses were treated as ordinal outcomes.

Results:

Because the age distribution differed between the 2018 and 2025 physician samples, additional ordinal logistic regression analyses were conducted with adjustment for age and sex. After adjustment, survey year was not significantly associated with responses to either the item assessing intention to use medical AI (likelihood-ratio χ² = 0.84, p = 0.360) or the item assessing the potential of medical AI to improve clinical efficiency (likelihood-ratio χ² = 0.66, p = 0.415; Supplementary Table S1). Age was significantly associated with both outcomes, whereas sex was not.

Discussion:

Among physicians, mean responses to the item assessing intention to use medical AI did not differ significantly between 2018 and 2025. In the unadjusted comparison, physicians surveyed in 2025 showed higher mean responses to the item assessing the potential of medical AI to improve clinical efficiency. However, this difference was no longer statistically significant after adjustment for age and sex, suggesting that differences in the age composition of the two physician samples may have contributed to the observed unadjusted difference. Thus, the present findings do not provide clear evidence that physicians’ perceptions of the efficiency benefits of medical AI increased independently of differences in sample characteristics.

Commet5:
No analysis of why intention to use didn't increase.

Response:

Thank you for this important comment. We agree that the original Discussion did not sufficiently examine why intention to use medical AI did not increase significantly. We have expanded the Discussion to consider several possible explanations, including the relatively autonomous form of medical AI described in the questionnaire, persistent concerns regarding safety, diagnostic responsibility, accountability, medical ethics, and legal regulation, and increased awareness of potential healthcare costs. We now emphasize that greater knowledge of or familiarity with AI may increase awareness of both its benefits and its risks and therefore may not directly translate into greater intention to use it. Because the questionnaire did not directly assess participants’ reasons for their intention to use or not use medical AI, we have also clarified that these explanations are hypotheses and added this point to the Limitations.

These revisions appear in the Discussion on page 14-15, lines 326–339, and in the Limitations on page 18, lines 441-444.

Discussion:

Several factors may explain why higher familiarity with medical AI did not translate into a significant increase in intention to use it. First, the questionnaire described a relatively autonomous form of medical AI that determines diagnoses and treatment plans without physician involvement. Greater exposure to general-purpose AI or physician-assistive systems may therefore not lead to greater willingness to use the more autonomous system described in this study. Second, increased knowledge may heighten awareness not only of potential benefits but also of issues related to safety, diagnostic responsibility, accountability, medical ethics, and legal regulation. Among members of the general public, the higher rating concerning possible increases in healthcare costs may also have restrained willingness to use medical AI. These considerations suggest that intention to use medical AI may depend on whether perceived benefits outweigh concerns regarding autonomy, risk, responsibility, and cost, rather than on familiarity or knowledge alone. However, because the questionnaire did not directly assess participants’ reasons for their intention to use or not use medical AI, these explanations should be regarded as hypotheses rather than conclusions demonstrated by the present data.

Limitations:

Sixth, the questionnaire did not directly assess the reasons underlying participants’ intention to use or not use medical AI. Therefore, the explanations proposed for the absence of a significant increase in intention to use are interpretive and should be examined directly in future studies using measures of perceived risk, trust, responsibility, autonomy, and cost concerns.

Please let us know if further clarification or revisions are needed.

Dear Reviewer [3] ,

We would like to thank you for your thoughtful and constructive comments. We have carefully revised the manuscript to address all the issues you raised. Below, we provide point-by-point responses to your comments, with explanations of how we have addressed each one in the revised manuscript.

----

Reviewer #3: I appreciate the invitation for this manuscript. The manuscript is well written, and the topic is of interest; however, I believe it is out of the scope of this journal. Here are my comments on the article below:

- Introduction:
1) The introduction is well-written in English and informative; however, it is too long. I recommend the authors to summarize the introduction in three paragraphs and less than 400 words.

Response:

Thank you for this helpful suggestion. We have revised the Introduction into three paragraphs and reduced it to fewer than 400 words. Redundant background information was removed, and the revised text now focuses on previous evidence, recent developments in medical AI, the remaining research gap, and the objective of the present repeated cross-sectional study.

The revised Introduction appears on pages 3, lines 74–103.

Background:

Artificial intelligence (AI) has increasingly been introduced into healthcare to support clinical diagnosis, treatment decision-making, and operational efficiency [1,2]. However, acceptance of medical AI has remained uncertain, particularly because patients and the public have expressed concerns regarding safety, accountability, and the role of AI in clinical care [3]. In Japan, government-led initiatives promoting the societal implementation of medical AI began around 2017, increasing the need to understand attitudes among both healthcare professionals and the general public. Against this background, Tamori et al. conducted a nationwide survey in 2018 and found that physicians were generally more positive about medical AI and reported greater intention to use it than members of the general public, although both groups expressed substantial concerns regarding legal responsibility and explainability [4].

Similar findings have been reported internationally. Surveys of patients and the public have shown that perceived usefulness may coexist with concerns regarding safety and accountability [5], while physicians have reported positive evaluations of the clinical usefulness of AI alongside concerns about legal responsibility and explainability [6]. Since the COVID-19 pandemic, healthcare digitalization has accelerated through the expansion of telemedicine and remote monitoring [7–11]. More recently, the emergence of large language models and generative AI has further increased public and professional exposure to AI technologies [12]. At the same time, ethical, legal, and transparency-related concerns have intensified, suggesting that acceptance depends not only on technical usefulness but also on trust and appropriate governance [13,14]. In addition, Yang et al. reported that although clinical decisions supported by generative AI were considered reasonable, physicians’ perceived competence and trust among peers could be adversely affected [15], indicating that social and psychological factors also shape acceptance.

These developments suggest that attitudes toward medical AI may have changed since 2018, particularly with respect to perceived usefulness, efficiency, reliability, legal concerns, and intention to use. However, previous studies have largely relied on cross-sectional surveys and have not examined changes using the same questionnaire across different time points. Therefore, the present study conducted a repeated cross-sectional survey in 2025 using the questionnaire originally administered by Tamori et al. in 2018. The objective was to compare item-level responses among physicians and members of the general public between 2018 and 2025 and to explore the underlying factor structure of attitudes toward relatively autonomous medical AI.

- Method:
1) please reference the textbox 1 in the text.

Response:

Thank you for pointing this out. We have added an explicit reference to Textbox 1 in the Methods section, where the questionnaire items are described.

The revision appears on page 4, lines 124–125.

Methods: **Questionnaire**

Textbox 1 presents the full wording of the 20 questionnaire items used to assess acceptance of medical AI.

2) Please also report why 300? what formula was used for calculation of sample size?

Response:

Thank you for this comment. No formal a priori sample size calculation was conducted for the 2025 survey, and no formula was used to determine the sample size. The target sample size of 300 physicians and 300 members of the general public was determined based on the available research budget and the feasibility of recruitment through the commercial online panel. We have clarified this point in the Methods and added the absence of an a priori sample size calculation as a limitation. These revisions appear in the Methods on page 4, lines 142–144, and in the Limitations on page 17, lines 420–421.

Methods:

No formal a priori sample size calculation was conducted. The target sample size of 300 physicians and 300 members of the general public was determined based on the available research budget and the feasibility of recruitment through the commercial online panel.

Limitations:

Second, no formal a priori sample size calculation was conducted, and the 2025 sample size was determined based on budgetary and feasibility considerations.

3) Lines 161-166 belong to method section rather than results.

Response:

Thank you for pointing this out. We have moved the methodological information in lines 161–166 to the Methods section and removed it from the Results. The Results section now reports only the survey dates, sample sizes, and participant characteristics.

4) please re-write the method section in a more detailed and segmented manner. (e.g., sample selection, ethical statement, questionnaire, ....)

Response:

Thank you for this helpful suggestion. We have substantially reorganized the Methods section using separate subheadings to improve clarity and reproducibility. The revised section is now divided into study design and definition of medical AI, questionnaire, participants and sample selection, survey procedures and ethical considerations, statistical analysis, and factor analysis. We also clarified participant recruitment, sample-size determination, questionnaire administration, item-level comparisons, and the age- and sex-adjusted sensitivity analyses, while removing redundant descriptions.

These revisions appear on pages 3–7, lines 105–193.

Methods:

**Study design and definition of medical AI**

This study used a repeated cross-sectional design to compare responses obtained in 2018 and 2025. The 2025 survey used the same questionnaire as the 2018 “Survey on Acceptance of the Use of Artificial Intelligence (AI) in Healthcare.” In both surveys, medical AI was defined as healthcare in which AI determines diagnoses and treatment plans without requiring physician involvement. This definition was retained unchanged to ensure comparability between the two survey years.

**Questionnaire**

The questionnaire consisted of two sections. The first covered respondents’ basic characteristics (e.g., sex, occupation, and age). The second measured acceptance of medical AI using 20 items developed with reference to the Unified Theory of Acceptance and Use of Technology (UTAUT) [16]. Because the UTAUT was originally designed for users of specific systems, and this study included members of the general public who were non-users, items assuming actual use experience or use frequency were excluded, and the wording was modified to assess cognitive evaluations based on hypothetical use. In addition, attitude and uneasiness were included, as previous studies have confirmed them to influence intention to use. All 20 items were rated on a five-point Likert scale (1 = strongly disagree, 5 = strongly agree). Only the item related to medical costs used a different scale (1 = decrease to 5 = increase). Some items (e.g., concerns about data leakage), were reverse-coded so that higher scores consistently indicated greater acceptance. Textbox 1 presents the full wording of the 20 questionnaire items used to assess acceptance of medical AI.

**Participants and sample selection**

Participants in both surveys were recruited from a commercial opt-in online research panel maintained by Rakuten Insight, Inc., Japan. Physician status was determined based on participants’ self-reported occupation and was not independently verified. Physician participants also self-reported their medical specialty by selecting one specialty category.

The 2018 survey initially included 600 members of the general public and 400 self-identified physicians. The general public sample was divided into six age groups (15–24, 25–34, 35–44, 45–54, 55–64, and ≥65 years), with 50 men and 50 women in each group. The physician sample consisted of participants aged ≥25 years and included 350 men and 50 women. Physicians were recruited regardless of clinical experience or medical specialty. One respondent who self-identified as a physician was excluded because the reported highest educational attainment was vocational school/junior college, which was inconsistent with the educational requirements for obtaining a medical license in Japan. The final 2018 analytical sample therefore consisted of 399 physicians and 600 members of the general public.

The 2025 survey included 600 participants, comprising 300 self-identified physicians and 300 members of the general public. No formal a priori sample size calculation was conducted. The target sample size of 300 physicians and 300 members of the general public was determined based on the available research budget and the feasibility of recruitment through the commercial online panel.

**Survey procedures and ethical considerations**

Before beginning the 2025 survey, participants were provided with information regarding the purpose of the study, the approximate completion time, the voluntary nature of participation, and the handling of their responses. Participants provided electronic consent before proceeding to the questionnaire. All responses were anonymous, and no personally identifiable information was collected. Participants received panel points upon completion of the survey. Information on sex, age, and highest educational attainment was collected as demographic characteristics. All analyses were conducted separately for physicians and members of the general public.

**Statistical analysis**

The 2018 and 2025 samples were treated as independent samples, and responses were compared separately for physicians and members of the general public.

For each item, the mean and standard deviation were calculated and compared between 2018 and 2025. Analyses were conducted separately for physicians and the general public using independent-samples t-tests. Effect sizes were calculated using Cohen’s d and corresponding 95% confidence intervals and interpreted according to Cohen’s (1988) criteria, with values of 0.2, 0.5, and 0.8 indicating small, medium, and large effects, respectively.

Because the age distribution differed between the 2018 and 2025 physician samples, additional ordinal logistic regression analyses were conducted as sensitivity analyses. Separate models were fitted for the items assessing intention to use medical AI and the perceived potential of medical AI to improve clinical efficiency. Survey year was included as the primary explanatory variable, with age and sex included as covariates. The questionnaire responses were treated as ordinal outcomes.

Factor analysis

Exploratory factor analysis (EFA) was conducted using the 2018 dataset to identify the factor structure underlying acceptance of medical AI. To examine the validity of the identified factor structure, confirmatory factor analysis (CFA) was then performed to evaluate the model’s fit to both the 2018 and 2025 datasets. Model fit was assessed using goodness-of-fit indices and inter-factor correlations separately for each survey year. Because the questionnaire items were ordinal in nature, estimation used diagonally weighted least squares (DWLS) method. Statistical analyses were conducted using R (version 4.5.2) and JMP, and CFA was performed using lavaan package.

Comparisons between the 2018 and 2025 surveys were conducted at the level of individual questionnaire items. The study did not compare latent factor means or composite scale scores across survey years.

- Results:
1) please report the specialty of physicians. different specialties may have different opinions, for example, a radiologist may appreciate the use of AI more than an internal medicine.

Response:

Thank you for this important comment. Medical specialty was self-reported by physician participants in both surveys, with each participant selecting one specialty category. We have clarified this point in the Methods and added the distribution of medical specialties by survey year in Supplementary Table S2. We have also summarized the principal specialty categories in the Results. These revisions appear in the Methods on page 4, lines 129–131, in the Results on page 7, lines 199–201, and in Supplementary Table S2.

Methods:

Physician status was determined based on participants’ self-reported occupation and was not independently verified. Physician participants also self-reported their medical specialty by selecting one specialty category.

Results:

The distribution of self-reported medical specialties among physicians in each survey year is presented in Supplementary Table S2.

Please let us know if further clarification or revisions are needed.
