# Supplementary material for: Changes in the acceptance of artificial intelligence in medicine among physicians and the general public in Japan: A repeated cross-sectional comparison of 2018 and 2025
Source: PLOS Digit Health. 2026 Oct 23;5(10):e0001769. doi: 10.1371/journal.pdig.0001769 (PMC13693883; doi:10.1371/journal.pdig.0001769)
Supplement: Peer review history

# Peer review: Decision Letter 0

**DOI:** 10.1371/journal.pdig.0001769.r001

**Authors:** Alaa Youssef, Laura Maaß

8 Jun 2026

Response to Reviewers Revised Manuscript with Track Changes Manuscript **Journal Requirements:**

1. Please amend your detailed Financial Disclosure statement. This is published with the article. It must therefore be completed in full sentences and contain the exact wording you wish to be published.

a. State the initials, alongside each funding source, of each author to receive each grant.

b. State what role the funders took in the study. If the funders had no role in your study, please state: “The funders had no role in study design, data collection and analysis, decision to publish, or preparation of the manuscript.”

c. If any authors received a salary from any of your funders, please state which authors and which funders.

**Additional Editor Comments (if provided):** **Reviewers' Comments:**

**Comments to the Author**

1. Does this manuscript meet PLOS Digital Health’s publication criteria?

Reviewer #1: Yes

Reviewer #2: Yes

Reviewer #3: No

**********

2. Has the statistical analysis been performed appropriately and rigorously?

Reviewer #1: Yes

Reviewer #2: Yes

Reviewer #3: Yes

**********

3. Have the authors made all data underlying the findings in their manuscript fully available (please refer to the Data Availability Statement at the start of the manuscript PDF file)?

The PLOS Data policy

Reviewer #1: Yes

Reviewer #2: Yes

Reviewer #3: Yes

**********

4. Is the manuscript presented in an intelligible fashion and written in standard English?

Reviewer #1: Yes

Reviewer #2: Yes

Reviewer #3: Yes

**********

Reviewer #1: - Introduction

- This is a study addressing a highly relevant question regarding the acceptance and attitudes towards the use of medical AI among physicians and the general public. The relevance of this question and the need for the study are well-articulated.

- I don’t think this study should be called a “seven-year comparative study (2018-2025)”, which sounds like a repeated cross-sectional study conducted every year for the seven years. Rather, it is a repeated cross-sectional study comparing 2018 and 2025. In a similar vein, I wouldn’t call it a trend study because only two time points are compared.

- Methods

- The definition of medical AI is quite restrictive, and this nuance and its potential impact on interpretation of the results should be mentioned in the Discussion. “Healthcare in which AI determines diagnoses and treatment plans without requiring physicians” are high-risk devices that require stringent regulatory approvals. In contrast, the increase in the exposure and knowledge regarding AI is likely to have been more on the general purpose health-related AI or AI-based assistive devices for physicians’ decision making. This nuance/discrepancy should be taken into account when interpreting the results and considering the implications of the results.

- Please report the sampling/recruitment method. I would like to see more details than the fact that it was conducted online by a company called Rakuten Insight. Methodological details may matter for the interpretation of the results.

- Results

- Table 1 and 2 can be combined. The combined table can retain the format of the current Table 2, and the rows for sex and age group can be added. Please report percentages so that is is easier to compare across doctor vs. public and across 2018 vs. 2025.

- Table 3 in the current form is not very informative because there is no meaningful target population that the sample (physician+public) represents. Instead, it might be more informative to have Table 3 to describe the difference between the physicians vs. public in 2018, and the difference between the physicians vs. public in 2025. These are only suggestions, and if these are not the authors’ questions, simply Table 3 can be deleted.

- All acronyms should be defined (SRMR, TLI, RMSEA, etc.).

- There are too many tables. More thought should be put into the presentations of the findings. For example, Table 7 and 10 can be combined. Some tables could be supplementary. There is no correct way but please try to improve the presentations.

- Discussion

- Please use the first paragraph to summarize and interpret the main findings of the study.

- There is no need to directly refer to the Results when discussing the results. You can delete phrases like “as shown in Table 4”.

Reviewer #2: This study examines changes in medical AI acceptance among Japanese physicians and the public over 7 years (2018–2025) using identical questionnaires. The core finding—that perceived usefulness remained high but intention to use did not significantly increase, while concerns persisted—is relevant for AI implementation policy. The study is methodologically sound with clear presentation, but suffers from largely confirmatory findings, suboptimal factor model fit, and lack of measurement invariance testing across survey years.

The 5-factor EFA model (Table 6) was abandoned after CFA showed high inter-factor correlations (r=0.92 between F1-F2) and poor discriminant validity. The simplified 3-factor model still shows RMSEA >0.10 in both years (2025: 0.106; 2018: 0.121), well above the 0.08 cutoff. Authors dismiss this as "some conceptual heterogeneity" (line 299), but this indicates poor model fit.

Authors state: "measurement invariance was not examined stepwise" (line 413). Without establishing configural, metric, and scalar invariance, comparing mean scores across 2018 vs. 2025 is not justified. This is a fundamental requirement for longitudinal comparisons [Vandenberg & Lance, 2000].

The rationale for collapsing F1, F2, F5 into F'1 is vague: "prioritized interpretability and structural stability" (line 288).

Age distribution differs markedly between years (Table 1): 2018 physicians skewed older (45-64), 2025 more balanced.

No analysis of why intention to use didn't increase.

Reviewer #3: I appreciate the invitation for this manuscript. The manuscript is well written, and the topic is of interest; however, I believe it is out of the scope of this journal. Here are my comments on the article below:

- Introduction:

1) The introduction is well-written in English and informative; however, it is too long. I recommend the authors to summarize the introduction in three paragraphs and less than 400 words.

- Method:

1) please reference the textbox 1 in the text.

2) Please also report why 300? what formula was used for calculation of sample size?

3) Lines 161-166 belong to method section rather than results.

4) please re-write the method section in a more detailed and segmented manner. (e.g., sample selection, ethical statement, questionnaire, ....)

- Results:

1) please report the specialty of doctors. different specialties may have different opinions, for example, a radiologist may appreciate the use of AI more than an internal medicine.

- Discussion: well written

- References: up to date

**********

what does this mean?). If published, this will include your full peer review and any attached files.

**Do you want your identity to be public for this peer review?**  If you choose “no”, your identity will remain anonymous but your review may still be made public.

For information about this choice, including consent withdrawal, please see our Privacy Policy

Reviewer #1: No

Reviewer #2: No

Reviewer #3: No

**********

**Figure resubmission:**

**Reproducibility:**

To enhance the reproducibility of your results, we recommend that authors of applicable studies deposit laboratory protocols in protocols.io, where a protocol can be assigned its own identifier (DOI) such that it can be cited independently in the future. Additionally, PLOS ONE offers an option to publish peer-reviewed clinical study protocols. Read more information on sharing protocols at https://plos.org/protocols?utm_medium=editorial-email&utm_source=authorletters&utm_campaign=protocols

# Author comment: Author response to Decision Letter 1

**DOI:** 10.1371/journal.pdig.0001769.r002

7 Jul 2026

Attachment Submitted filename: Response to Reviewers.docx

# Peer review: Decision Letter 1

**DOI:** 10.1371/journal.pdig.0001769.r003

**Authors:** Alaa Youssef, Laura Maaß

13 Sep 2026

Changes in the Acceptance of Artificial Intelligence in Medicine Among Physicians and the General Public in Japan: A Repeated Cross-Sectional Comparison of 2018 and 2025

PDIG-D-26-00290R1

Dear Mr. Ogasawara,

We are pleased to inform you that your manuscript 'Changes in the Acceptance of Artificial Intelligence in Medicine Among Physicians and the General Public in Japan: A Repeated Cross-Sectional Comparison of 2018 and 2025' has been provisionally accepted for publication in PLOS Digital Health.

Before your manuscript can be formally accepted you will need to complete some formatting changes, which you will receive in a follow-up email from a member of our team.

Please note that your manuscript will not be scheduled for publication until you have made the required changes, so a swift response is appreciated.

IMPORTANT: The editorial review process is now complete. PLOS will only permit corrections to spelling, formatting or significant scientific errors from this point onwards. Requests for major changes, or any which affect the scientific understanding of your work, will cause delays to the publication date of your manuscript.

If your institution or institutions have a press office, please notify them about your upcoming paper to help maximize its impact. If they'll be preparing press materials, please inform our press team as soon as possible -- no later than 48 hours after receiving the formal acceptance. Your manuscript will remain under strict press embargo until 2 pm Eastern Time on the date of publication. For more information, please contact digitalhealth@plos.org.

Thank you again for supporting Open Access publishing; we are looking forward to publishing your work in PLOS Digital Health.

Best regards,

Laura Maaß, PhD

Academic Editor

PLOS Digital Health

***********************************************************

**Additional Editor Comments (if provided):**

**Reviewer Comments (if any, and for reference):**

Reviewer's Responses to Questions

**Comments to the Author**

Reviewer #1: All comments have been addressed

Reviewer #2: All comments have been addressed

Reviewer #3: All comments have been addressed

**********

publication criteria?

Reviewer #1: Yes

Reviewer #2: Yes

Reviewer #3: No

**********

3. Has the statistical analysis been performed appropriately and rigorously?

Reviewer #1: Yes

Reviewer #2: Yes

Reviewer #3: Yes

**********

4. Have the authors made all data underlying the findings in their manuscript fully available (please refer to the Data Availability Statement at the start of the manuscript PDF file)?

The PLOS Data policy

Reviewer #1: Yes

Reviewer #2: Yes

Reviewer #3: Yes

**********

5. Is the manuscript presented in an intelligible fashion and written in standard English?

Reviewer #1: Yes

Reviewer #2: Yes

Reviewer #3: Yes

**********

Reviewer #1: I have no additional comments.

Reviewer #2: The authors have responded carefully and have materially improved the manuscript. They now describe the repeated cross-sectional design, opt-in panel recruitment, autonomous-AI wording, absence of an a priori sample-size calculation, physician self-identification, poor CFA fit, and lack of measurement invariance more transparently. The title and principal conclusions are appropriately moderated. Nevertheless, the factor-analysis section remains prominent despite an inadmissible/improper solution and poor fit.

The three-factor model has poor RMSEA (>0.10), several weak loadings, a negative loading for Q20, and an apparent standardized loading >1 for Q18 in 2018. Combining factors after inspecting the CFA is data-driven and does not validate the new structure. This may represent an improper solution and does not support substantive claims about coherent dimensions.

Minor comments

- Report the ethics determination more precisely, including the governing institution/policy or exemption determination; “IRB is not required in Japan” is too broad.

- Cronbach’s alpha for all 20 heterogeneous items is difficult to interpret and should not be described as maintained reliability of one scale.

- Correct “physicians responses” to “physicians’ responses” and standardize table titles/spacing.

- Verify that the OSF deposit contains code, codebook, item coding (including reverse coding), and both datasets.

Reviewer #3: I appreciate the authors response. I have no further comments for this manuscript.

**********

what does this mean?). If published, this will include your full peer review and any attached files.

**Do you want your identity to be public for this peer review?**  If you choose “no”, your identity will remain anonymous but your review may still be made public.

For information about this choice, including consent withdrawal, please see our Privacy Policy

Reviewer #1: No

Reviewer #2: No

Reviewer #3: No

**********
